# Association Between White Blood Cells at Baseline and Treatment Failure of MTX for Ectopic Pregnancy

**DOI:** 10.3389/fmed.2021.722963

**Published:** 2021-09-10

**Authors:** Si Chen, Xiao-Feng Chen, Pin Qiu, Yan-Xi Huang, Gao-Pi Deng, Jie Gao

**Affiliations:** ^1^The First Clinical Medical College, Guangzhou University of Chinese Medicine, Guangzhou, China; ^2^Department of Gynecology, First Affiliated Hospital of Guangzhou University of Chinese Medicine, Guangzhou, China

**Keywords:** ectopic pregnancy, methotrexate, white blood cell count, treatment outcome, China

## Abstract

**Purpose:** The aim of this study was to evaluate white blood cell (WBC) count as a risk factor related to methotrexate (MTX) treatment failure in patients with ectopic pregnancy (EP).

**Methods:** A total of 236 women diagnosed with EP and treated with a single dose of MTX were included. The exposure variable was WBC count at baseline, and the outcome was MTX treatment outcome. Both a multivariate binary logistics regression model and subgroup analysis were performed to evaluate the association between WBC and MTX non-response.

**Results:** WBC count was associated with the risk of treatment failure, and the odds ratio (OR) in different multivariate models was stable [minimally adjusted model: OR 1.2, 95% confidence interval (CI): 1.0–1.3, *p* = 0.008; fully adjusted model: OR 1.2, 95% CI: 1.0–1.4, *p* = 0.026]. For WBCs in group T3 (>8.9 × 109/L), the association between WBC count and treatment failure was significant (minimally adjusted model: OR: 2.0, 95% CI: 1.0–3.8, *p* = 0.050; fully adjusted model: OR: 2.2, 95% CI: 1.1–5.6, *p* = 0.034). Subgroup analysis showed that in participants with regular menstruation (OR 1.1, 95% CI: 1.0–1.3), WBC count was significantly different from irregular menstruation (OR 1.8, 95% CI: 1.2–2.8); *p* for interaction was 0.031.

**Conclusions:** We found a reliable and non-linear relationship between WBC count and MTX treatment failure for EP.

## Introduction

Ectopic pregnancy (EP) refers to the implantation of fertilized eggs outside the uterine cavity. Based on the 2018 American College of Obstetricians and Gynecologists (ACOG) guidelines, EP occurs in about 2% of all pregnancies and causes 2.7% of all pregnancy-related deaths (1). It is one of the main causes of hemorrhagic shock and death in pregnant women (2). With the recent increase in reproductive tract infections, tubal surgery, and assisted reproductive technology, EP has shown a progressive increase that imposes not only an economic burden but also a psychological burden on individuals, families, and society. Treatments for EP mainly include surgery and drug therapy. Compared with surgical treatment, drug therapy has the advantages of avoiding surgical complications and iatrogenic trauma as well as preserving the fallopian tubes (FT) to offer a higher chance of conception for those with fertility requirements. Drug therapy is a non-invasive, effective, safe, and low-cost therapeutic method (3). The common therapeutic drug for EP is methotrexate (MTX) (1); however, the success rate of MTX treatment in EP fluctuates between 65 and 95% (4–6). Failure of treatment can lead to rupture of the ectopic mass, rapid and continuous bleeding, and even life-threatening shock. Therefore, it is still necessary to find a predictive factor for MTX treatment, so that clinicians can evaluate the condition of EP patients more accurately and comprehensively and therefore choose more suitable treatment options for patients, in order to avoid the occurrence of critical conditions.

Infection is a substantial risk factor for EP. Histological and molecular studies have demonstrated that inflammation occurs at the site of EP (7). A previous study also indicated that inflammation-related indicators are significantly higher in patients with EP than in those with a normal intrauterine pregnancy during the same period (8).

Some studies have also found an association between inflammation-related indicators and treatment. Several inflammation-related indicators can be related to the efficacy of MTX, such as neutrophil–lymphocyte ratio (NLR), platelet–lymphocyte ratio (PLR), platelet distribution width (PDW), platelet mean volume (MPV), and platelet count (PLT) (9–11). White blood cell (WBC) count, as the most common and important test index for evaluating inflammation in clinical practice, is often measured for each patient on the first day of admission. This study set out to address the association between WBC count and the efficacy of MTX in the treatment of ectopic pregnancy because current data are sparse and conflicting. Furthermore, due to the Third Child Policy in China, the incidence of ectopic pregnancies is likely to rise; however, there has been a lack of relevant analysis based on Chinese population data to assess the association between inflammatory-related indicators and the efficacy of MTX for EP treatment.

We therefore performed a retrospective cohort study at the First Affiliated Hospital of Guangzhou University of Traditional Chinese Medicine, Guangdong Province, China, aiming to estimate the association between baseline WBC counts and the MTX treatment outcome in EP patients and to investigate whether WBC count could be a potential predictive factor for MTX treatment outcome.

## Materials and Methods

### Study Participants

This retrospective cohort study based on 236 patients was conducted at the First Affiliated Hospital of Guangzhou University of Traditional Chinese Medicine, Guangzhou City, China, from June 2016 to February 2019. The ethics committee approved the study [Approval No. ZYYECK (2018)157]. Baseline demographic characteristics were documented in the electronic record system (EDC). The diagnostic criteria of EP were based on the ACOG guidelines (1).

The inclusion and exclusion criteria in the study were as follows. Specific inclusion criteria are as follows: (1) all patients presented with vaginal bleeding or abdominal pain during early pregnancy; (2) serum β-HCG values of the patients were <4,500 IU/l; (3) transvaginal ultrasound may have identified EP but not intrauterine pregnancy. When an intrauterine or ectopic pregnancy was not identified, serial serum β-HCG measurements were performed to identify EP; (4) patients had an unruptured mass and stable hemodynamics; (5) patients who selected MTX treatment were informed of the precautions, and they closely adhered to the advice given. Exclusion criteria were (1) unstable hemodynamics; (2) patients preferred surgical management or expectant management; (3) patients were diagnosed with non-tubal pregnancy; (4) those treated with a double dose of MTX; and (5) absolute contraindications to MTX treatment, including intrauterine pregnancy, testimony of immunodeficiency, anemia of moderate to severe extent, thrombocytopenia or leukopenia, allergy to MTX, active peptic ulcer, active lung disease, hepatic dysfunction, renal dysfunction, and breastfeeding.

### Measurement of WBC Count

Baseline WBC counts of all patients as measured on the day of hospitalization were considered as the exposure variable. Baseline WBC count was taken as a continuous variable and categorical variable (tertile), while aiming to evaluate a stable connection between WBC count and MTX treatment of EP. We collected 2 ml of venous blood from each patient and placed it in an EDTA-containing anticoagulant tube. The blood samples were delivered to the hospital laboratory and analyzed by an automatic hematology analyzer (BM831, Baolingman Sunshine Technology Co. Ltd., Shenzhen, China).

### Outcome Variable Definition

MTX treatment failure was considered as the outcome variable. According to the ACOG guidelines (1), MXT treatment failure was defined as serum β-HCG levels that plateaued or increased between day 4 and day 7, or the patients presented with acute abdominal pain, drop in blood pressure, or even shock because of a ruptured ectopic pregnancy mass when admitted for emergency surgery.

### Other Covariates in the Study

The inclusion standard of covariates for fully adjusted model performance was based on previous studies regarding the risk factors of MTX treatment failure with EP (12–17). Consequently, the following covariates were included in this study: age, BMI, smoking history, gravidity, history of spontaneous abortion, history of infertility, history of ectopic pregnancy, history of pelvic inflammation, gestational age, abdominal, vaginal bleeding, baseline β-HCG, neutrophils percentage (NEU%), hemoglobin (HGB), platelets (PLT), ectopic mass size, pelvic effusion, and the presence of yolk sac.

### Treatment Protocol

Based on the relevant clinical guidelines (1), patients included in this study received MTX treatment in a single-dose protocol; the dose was an intramuscular injection of 50 mg/m^2^ of body surface area. The day when EP patients received MTX was considered as day 1. In order to evaluate the condition, serum β-HCG levels were measured on day 4 and day 7, and the ultrasound was performed on day 7.

### Statistical Analysis

We compared variables between the successful treatment group and the failure group. Categorical variables were shown as a percentage. In this study, one-way ANOVA was used to determine any statistical differences between the means and proportions of the groups; the Kruskal–Wallis H test was used for skewed distribution; and the chi-square test was used for categorical variables. Moreover, univariate linear regression model was performed to assess the association between WBC count and MTX treatment failure in EP. In addition, as described in the STROBE statement (18), we performed non-adjusted and multivariate adjusted models simultaneously to assess the association between WBC count and MTX treatment failure. According to the guidelines, covariates which were included in the model met the following requirement: the matched odds ratio changed by at least 10% when adding the covariants in the model (19). Furthermore, subgroup analysis was performed using hierarchical linear regression models. Interactions and modifications of subgroups were assessed by the likelihood ratio test. All analyses were performed using the statistical software packages R and EmpowerStats (http://www.empowerstats.com/cn/). *p* < 0.05 (two-sided) was regarded statistically important.

## Results

### Patient Selection

**Figure 1:**
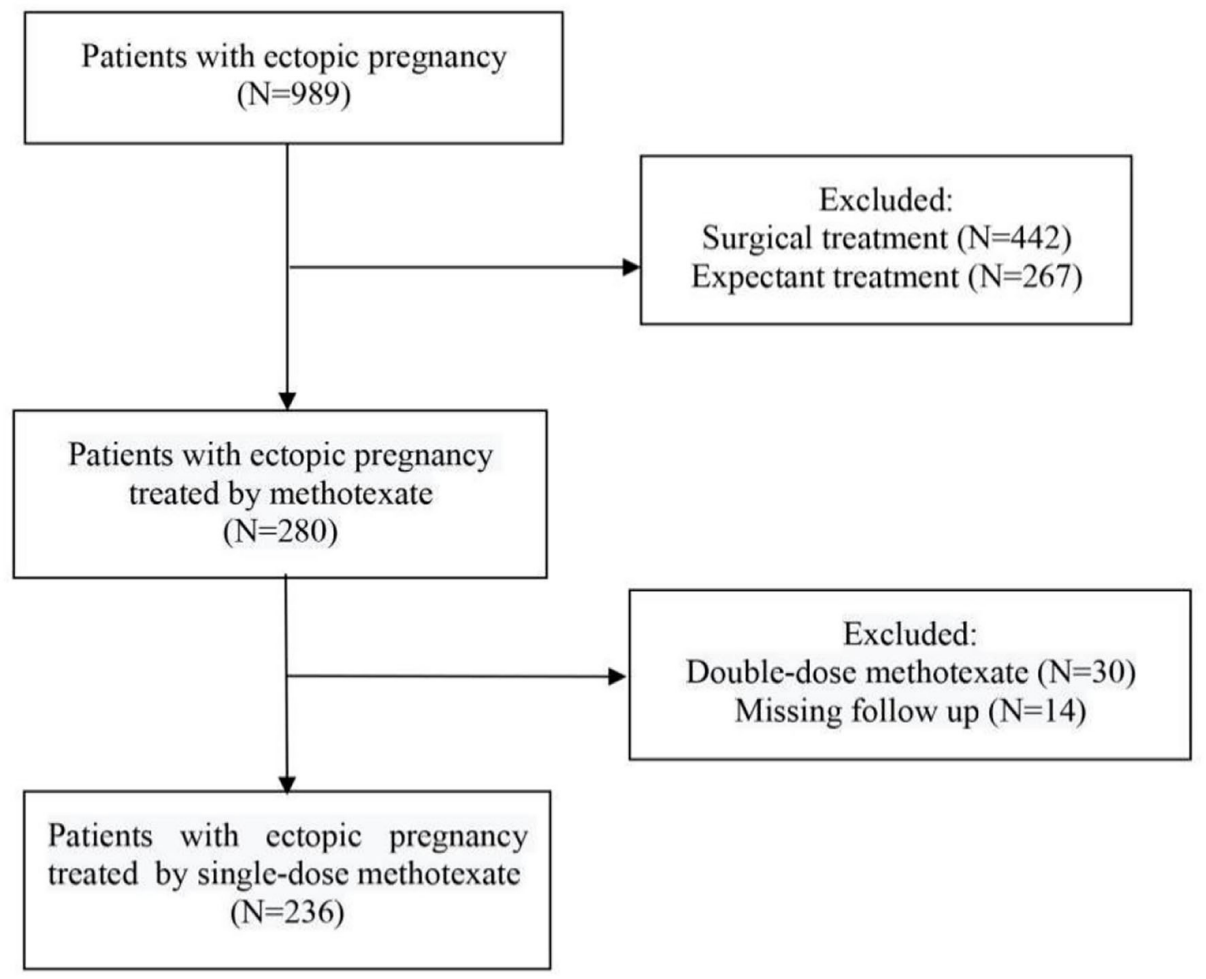
Study flowchart. This figure shows the procedure of patient selection.

A total of 989 patients diagnosed with EP were initially included in this study. Among them, 442 patients treated with surgery and 267 patients treated with expectant management were excluded. Among the remaining 280 patients, we excluded 44 patients because 30 patients were treated with double-dose MTX and 14 patients lacked relevant clinical data. Consequently, 236 patients met the inclusion and exclusion criteria of this study and were included as participants (Figure 1).

### Baseline Characteristics of Participants

The baseline characteristics of patients with EP are shown in Table 1. We compared baseline characteristics among the treatment success group and failure group and found that there were no statistically significant differences in age, BMI, gestational age, days of vaginal bleeding, days of abdominal pain, NEU%, HGB, PLT, ectopic mass size, pelvic effusion, gravidity, history of natural abortion, history of ectopic pregnancy, regular menstruation, or history of infertility. Compared with the treatment success group, patients had a significantly higher log10 baseline β-HCG, incidence of history of pelvic inflammation, and the presence of a yolk sac.

**Table 1:** Baseline characteristics of participants.

**Treatment outcome**	**Success**	**Failure**	***p*-value**
No. of participants	198	38	
Age (years, mean ± SD)	30.5 ± 5.5	30.4 ± 5.6	0.675
BMI (kg/m^2^, mean ± SD)	19.7 ± 5.8	20.1 ± 4.8	0.557
Gestational age z (days, mean ± SD)	48.5 ± 11.5	46.2 ± 11.3	0.142
Days of vaginal bleeding [days, median (min, max)]	10.0 (0.0–43.0)	7.0 (0.0–49.0)	0.106
Days of abdominal pain [days, median (min, max)]	1.0 (0.0–43.0)	1.0 (0.0–49.0)	0.193
WBC (× 10^9^/l, mean ± SD)	7.3 ± 2.1	8.1 ± 2.6	0.010
NEU% [%, median (min, max)]	65.9 (40.2 90.5)	67.3 (6.6 89.7)	0.162
HGB (g/l, mean ± SD)	122.8 ± 10.8	123.2 ± 10.9	0.769
PLT (× 10^9^/l, mean ± SD)	246.5 (54.6)	246.9 (53.1)	0.863
log10 baseline β-HCG [IU/l, median (min, max)]	2.9 (1.7–3.8)	3.2 (2.0–4.2)	<0.001^*^
Ectopic mass size [mm, median (min, max)]	24.0 (0.0–68.0)	24.0 (0.0–81.0)	0.498
Pelvic effusion [ml, median (min, max)]	19.0 (0.0–92.0)	18.5(0.0–95.0)	0.355
Gravidity (*n*, mean ± SD)	3.2 ± 1.6	3.1 ± 1.7	0.620
History of natural abortion (*n*, %)			0.088
No	121 (84.0%)	69 (75.0%)	
Yes	23 (16.0%)	23 (25.0%)	
History of ectopic pregnancy (*n*, %)			0.182
No	117 (81.2%)	68 (73.9%)	
Yes	27 (18.8%)	24 (26.1%)	
Menstrual regularity (*n*, %)			0.510
No	25 (17.4%)	13 (14.1%)	
Yes	119 (82.6%)	79 (85.9%)	
History of infertility (*n*, %)			0.621
No	134 (93.1%)	84 (91.3%)	
Yes	10 (6.9%)	8 (8.7%)	
History of pelvic inflammation (*n*, %)			<0.001^*^
No	129 (89.6%)	57 (62.0%)	
Yes	15 (10.4%)	35 (38.0%)	
Yolk sac (*n*, %)			0.006
No	130 (90.9%)	71 (78.0%)	
Yes	13 (9.1%)	20 (22.0%)	

*BMI, body mass index; WBC, white blood cell; NEU, neutrophils; HGB, hemoglobin; PLT, platelets*.

*Statistical analysis: Kruskal–Wallis H (skewed distribution) test and chi-square tests (categorical variables) were used to determine any statistical differences between the means and proportions of the groups*.

* *indicates that P value < 0.05*.

### Univariate Analysis

The results of univariate analysis are shown in Table 2. These results showed that WBC count (OR: 1.2, 95% CI:1.0–1.3), history of pelvic inflammation (OR: 5.3, 95% CI:2.7–10.4), HCG (OR: 1.0, 95% CI:1.0–1.0), and presence of yolk sac (OR: 2.8, 95% CI:1.3–6.0) were risk factors of MTX treatment failure in EP patients. We also found that age, BMI, gravidity, history of natural abortion, history of ectopic pregnancy, history of infertility, menstrual regularity, gestational age, days of vaginal bleeding, days of abdominal pain, NEU%, HGB, PLT, ectopic mass size, and pelvic effusion were not associated with treatment failure.

**Table 2:** Results of univariate analysis.

	**Statistics**	**Effect size (β)**	***p-*value**
Age (y)	30.5 ± 5.5	1.0 (0.9, 1.0)	0.865
BMI	19.9 ± 5.4	1.0 (1.0, 1.1)	0.556
Gravidity	3.2 ± 1.6	1.0 (0.8, 1.1)	0.793
**History of natural abortion**			
No	46 (19.5%)	Ref	
Yes	190 (80.5%)	0.6 (0.3, 1.1)	0.090
**History of ectopic pregnancy**			
No	185 (78.4%)	Ref	
Yes	51 (21.6%)	1.5 (0.8, 2.9)	0.183
**History of infertility**			
No	218 (92.4%)	Ref	
Yes	18 (7.6%)	1.3 (0.5, 3.4)	0.622
**History of pelvic inflammation**			
No	186 (78.8%)	Ref	
Yes	50 (21.2%)	5.3 (2.7, 10.4)	<0.001
**Menstrual regularity**			
No	38 (16.1%)	Ref	
Yes	198 (83.9%)	1.3 (0.6, 2.6)	0.511
Gestational age	47.6 ± 11.4	1.0 (1.0, 1.0)	0.146
Days of vaginal bleeding	10.4 ± 9.8	1.0 (1.0, 1.0)	0.162
Days of abdominal pain	3.8 ± 6.5	1.0 (0.9, 1.0)	0.343
WBC	7.6 ± 2.3	1.2 (1.0, 1.3)	0.012
NEU%	66.4 ± 10.9	1.0 (1.0, 1.0)	0.252
HGB	123.0 ± 10.8	1.0 (1.0, 1.0)	0.784
PLT	246.6 ± 53.9	1.0 (1.0, 1.0)	0.946
HCG	1,669.4 ± 1,998.5	1.0 (1.0, 1.0)	<0.001
Ectopic mass size	26.5 ± 16.4	1.0 (1.0, 1.0)	0.865
**Yolk sac**			
No	201 (85.9%)	1.0	
Yes	33 (14.1%)	2.8 (1.3, 6.0)	0.007
Pelvic effusion	20.2 ± 23.2	1.0 (1.0, 1.0)	0.391

*BMI, body mass index; WBC, white blood cell; NEU, neutrophils; HGB, hemoglobin; PLT, platelets*.

*Statistical analysis: one-way ANOVA test was used to determine any statistical differences between the means and proportions of the groups*.

### Results of the Unadjusted and Adjusted Models

A univariate linear regression model was used to assess the associations between WBC count and MTX treatment failure. Consequently, we performed the crude, minimally adjusted, and fully adjusted models shown in Table 3. In the crude model, WBC count showed a positive association with treatment failure (β = 1.2, 95% CI: 1.0–1.3, *p* = 0.012). In the minimally adjusted model (adjusted age and BMI), the result was similar (β = 1.2, 95% CI: 1.0–1.3, *p* = 0.008). Furthermore, after adjustment of more covariants, including age, BMI, history of natural abortion, menstrual regularity, history of pelvic inflammation, PLT, and yolk sac, the association was still stable (β = 1.2, 95% CI: 1.1–1.4, *p* = 0.006).

**Table 3:** Relationship between WBC count and MTX treatment failure in different models.

**Variable**	**Crude model** **(β, 95% CI, *p*)**	**Minimally adjusted model (β, 95% CI, *P*)**	**Fully adjusted model (β, 95% CI, *P*)**
WBC	1.2 (1.0, 1.3) 0.012	1.2 (1.0, 1.3) 0.008	1.2 (1.1, 1.4) 0.006
**WBC (tertile)**			
T1 (≤5.9 × 10^9^/l)	Ref	Ref	Ref
T2 (5.9 × 109/l−8.9 × 109/l)	1.1 (0.5, 2.1) 0.866	1.0 (0.5, 2.0) 0.920	1.4 (0.7, 3.0) 0.348
T3 (>8.9 × 109/l)	1.9 (1.0, 3.7) 0.042	2.0 (1.0, 3.8) 0.050	2.2 (1.1, 5.6) 0.034
*p* for trend	<0.001	0.001	<0.001

*Crude model: we did not adjust other covariants*.

*Minimally adjusted model: we adjusted age and BMI*.

*Fully adjusted model: we adjusted age, BMI, history of natural abortion, menstrual regularity, history of pelvic inflammation, PLT, and yolk sac*.

*CI, confidence interval; Ref, reference*.

*Statistical analysis: multiple regression analysis was performed to evaluate the association between WBC count and treatment failure both as a continuous variable and as a categorical variable. The covariance adjusted in the fully adjusted model was determined by the following principle: when added to this model, the matched odds ratio was changed by at least 10%*.

To perform a sensitivity analysis, we transformed WBC count to a categorical variable (tertile). It should be noted that with patients in the highest tertile of baseline WBC count (>8.9 × 109/l), a significant effect was observed between an increase in WBCs and MTX treatment failure (crude model: β = 1.9, 95% CI: 1.0–3.7, *p* = 0.042; minimal model: β = 2.0, 95% CI: 1.0–3.8, *p* = 0.050; fully adjusted model: β = 2.2, 95% CI: 1.1–5.6; *p* = 0.034). When patients' WBCs were under 8.9 × 109/l, the association was not significant. The trend of WBCs in different adjustment strategies demonstrated that this was an independent risk factor for MTX treatment failure in EP. Furthermore, when WBCs were >8.9 × 109/l (T3 group), the increased WBC count was associated with a poor MTX treatment outcome.

### Results of Subgroup Analysis

**Figure 2:**
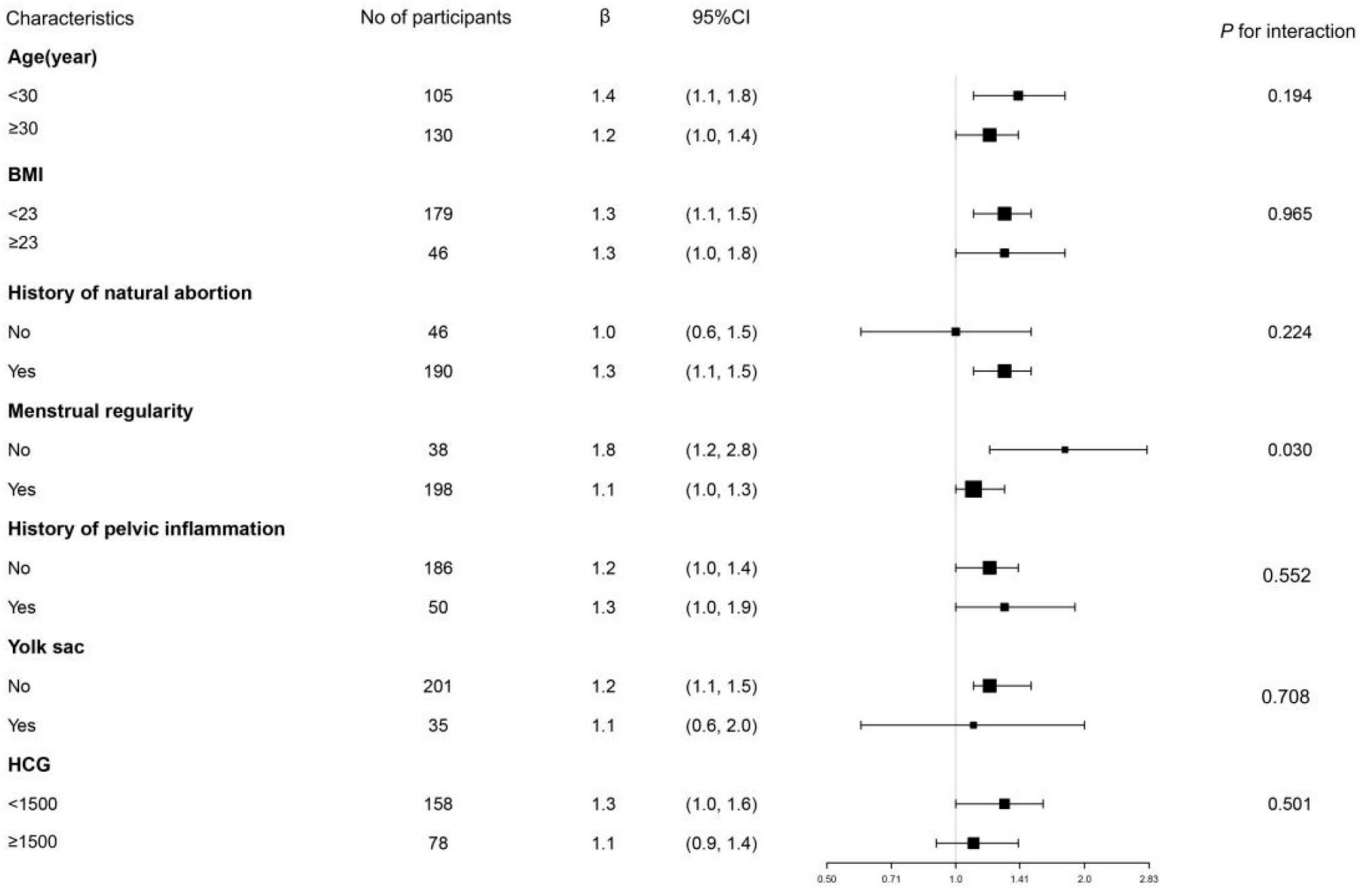
Tree View of subgroup analysis of WBC count and MTX treatment failure the tree diagram displays the results of subgroup analysis. It indicates that the test for interaction was significant for menstrual regularity (*p* for interaction = 0.031). In EP patients with regular menstruation, OR was 1.1 (1.0–1.3). In patients with irregular menstruation, OR was 1.8 (1.2–2.8). *p* for interaction was not significant in age, BMI, history of natural abortion, history of pelvic inflammation, yolk sac, and baseline HCG.

In this study, we also performed a subgroup analysis as displayed in Table 4 and Tree View as displayed in Figure 2. Subgroup analyses were performed by age (<30, ≥30 years), BMI (<23, ≥23), history of natural abortion, menstrual regularity, history of pelvic inflammation, yolk sac, and serum β-HCG (<1,500 IU/l, ≥1,500 IU/l). We found that the *p*-value of interaction was significant for menstrual regularity (p for interaction = 0.003). The *p*-values for interaction were not significant for age, BMI, history of natural abortion, menstrual regularity, history of pelvic inflammation, yolk sac, and β-HCG (p for interaction = 0.194, 0.965, 0.224, 0.552, 0.708, and 0.501). We further observed the interaction between menstrual regularity and WBC count. In patients with irregular menstruation, OR was 1.8 (1.2–2.8), while in patients with menstrual regularity, OR was 1.1 (1.0–1.3). OR values between regular and irregular menstruation were significantly different (as detected by the log-likelihood ratio test).

**Table 4:** Results of subgroup analysis and interaction analysis.

**Characteristic**	**No of participants**	**Effect size (95% CI)**	***p* for interaction**
**Age (years)**			
<30	105	1.4 (1.1, 1.8)	0.194
≥30	130	1.2 (1.0, 1.4)	
**BMI**			
<23	179	1.3 (1.1, 1.5)	0.965
≥23	46	1.3 (1.0, 1.8)	
**History of natural abortion**			
No	46	1.0 (0.6, 1.5)	0.224
Yes	190	1.3 (1.1, 1.5)	
**Menstrual regularity**			
No	38	1.8 (1.2, 2.8)	0.030
Yes	198	1.1 (1.0, 1.3)	
**History of pelvic inflammation**			
No	186	1.2 (1.0, 1.4)	0.5519
Yes	50	1.3 (1.0, 1.9)	
**Yolk sac**
No	201	1.2 (1.1, 1.5)	0.7079
Yes	35	1.1 (0.6, 2.0)	
**B-HCG**
<1500	158	1.3 (1.0, 1.6)	0.501
≥1500	78	1.1 (0.9, 1.4)	

*The above model was adjusted for age, BMI, history of natural abortion, menstrual regularity, history of pelvic inflammation, PLT, and yolk sac*.

*In each case, the model was not adjusted for the stratification variable*.

*Statistical analysis: the subgroup analyses were performed using stratified linear regression models. The modification and interaction of subgroups were inspected using the likelihood ratio test*.

## Discussion

This retrospective cohort study revealed an association between WBC count and MTX treatment failure with EP, after adjustment of potential covariants, showing that WBC count was an independent risk factor for MTX treatment failure in EP. Moreover, when converting WBC count into a categorical variable, its association with poor MTX treatment outcome still existed in group T3 (>8.9 × 109/l). This indicated that the association was non-linear, which is the highlight of the current study. In addition, we further performed subgroup analysis and found a significant interactive effect of menstrual regularity (*p* = 0.03).

These findings may have a positive impact on the early prediction of MTX efficacy in the treatment of EP. A WBC count and a routine checkup on admission should enable clinicians to choose more appropriate treatment options for patients with EP, thus avoiding serious complications and unnecessary economic loses.

Studies regarding associations between WBC counts and EP treatment outcome were previously limited and conflicting. We conducted a PubMed search simultaneously using “[(white blood cell [All Fields]) OR (leukocytes)] AND (ectopic pregnancy) AND (treatment).” Fifty-five related studies were retrieved from the database as of October 2020. Among these studies, only one was related to our study, which was inconsistent with our findings. The latter study (*n* = 153) suggested that there was no statistically significant difference in WBC counts between the MTX treatment group and the surgical treatment group (20). However, there were several disadvantages to this research, which led to the unreliability of the outcome. For example, the researchers conducted univariate analysis (Kruskal–Wallis test) which showed no statistically significant difference between the groups in terms of WBCs; in addition, there were no further multivariate analyses or adjustment of any covariants. Moreover, the lack of statistical significance in univariate analysis did not truly reflect the effect of WBC count, which may have been masked by the effects of other variables.

In the current study, we not only adjusted the potential covariants reported in the previous related studies that may affect MTX treatment outcome but also converted WBC count into a categorical variable and found that WBCs in the T3 group (>8.9 × 10^9^/l) were positively associated with poor MTX treatment outcome. Furthermore, according to the STROBE statement (18), aiming to make better use of data to reveal the underlying truth, we performed subgroup analysis and found an interaction effect on menstrual regularity. This suggested that clinicians should pay more attention to the levels of WBCs in patients with irregular menstruation.

Other inflammation-related serological indicators were found to be associated with treatment of EP. Cekmez et al. (11) report that MPV and NLR levels were higher in a successful MTX treatment group than in a failure group, suggesting that higher MPV and NLR are protective factors for success with single-dose MTX treatment. Kanmaz et al. (9) report that NLR could be used to evaluate the success of single-dose MTX administration in treating EP. Another study (9) found that both the levels of NLR and PLR were significantly higher in an FT rupture group compared with the levels of NLR and PLR in a non-rupture EP group, which suggests that inflammation markers are predictors for the risk of FT rupture. These previous studies only conducted univariate analysis while potential confounding factors were not taken into consideration. We consider that, compared to these previous studies, our study adhered more strictly to the STROBE guidelines (21). Furthermore, because we performed logistic regression analysis using WBC count as a continuous variable and categorical variable, and also performed a subgroup analysis, our results are comparatively more accurate.

EP is caused by the retention of embryos in the FT owing to impaired embryo-FT transport and alteration in the FT environment which allows an embryo to implant (22). Inflammation is one of the most important factors to affect tubal transport and the tubal microenvironment (23, 24). According to several studies, inflammation changes the molecular signals from FT tissues, induces pro-inflammatory phenotypes, induces the upregulation of cytokines that promote embryo receptivity, and provides many preimplantation signals that are recognized by blocked embryos, which results in EP (22, 25). Studies have also shown an increased expression of inflammation-related cytokines at the site of EP, such as interleukin (IL)-1 (26), IL-6 (25), IL-8 (25), secretory leukocyte protease inhibitor (SLPI) (8), elastase inhibitor (8), leukemia inhibitory factor (LIF) (27), and β-catenin (28).

Extravasation of WBCs is an important feature of the inflammatory response and is related to the development of EP. During pregnancy, WBCs play an important role in suppressing the mother's immune response to a semiallogenic fetus and limiting the invasiveness of the trophoblast (29). Embryo implantation is thought to trigger the recruitment of WBCs (30, 31), and *in vitro* experiments have also found an increase in the number of WBCs in the decidua tissue of women with EP (32). An additional clinical study (9) found that the total numbers of WBCs and monocytes found for ectopic pregnancies were significantly higher than those of normal intrauterine pregnancies during the same period, suggesting that the increase in WBC count is related to the development of EP. Combined with our finding that WBC count was positively associated with poor MTX treatment outcome, we subsequently inferred that the higher the WBC count, the deeper the embryo had implanted, and this resulted in a poorer treatment effect. However, the specific underlying mechanism between WBC count, inflammation, and treatment outcome of EP needs further study.

Several limitations should be considered in our study. First, all participants were recruited in China, which suggests prudence in extrapolating these results to populations with different demographic characteristics. Second, related prospective studies should be performed to strengthen the power of the evidence.

## Data Availability Statement

The original contributions presented in the study are included in the article/Supplementary Material, further inquiries can be directed to the corresponding author/s.

## Ethics Statement

The studies involving human participants were reviewed and approved by the ethics committee of First Affiliated Hospital of Guangzhou University of Chinese Medicine [No. ZYYECK(2018)157]. The data are anonymous, and the requirement for informed consent was therefore waived. Written informed consent for participation was not required for this study in accordance with the national legislation and the institutional requirements.

## Author Contributions

G-PD, SC, and X-FC: study concept and design. SC and X-FC: drafting of the manuscript and statistical analysis. X-FC, PQ, and Y-XH: data collection, clinical management, and record keeping. JG and G-PD: manuscript reviewing. All authors contributed to the article and approved the submitted version.

## Funding

This study was supported by the Scientific Research Team Training Project of GZUCM (No. 2019KYTD202), Deng Gaopi's Famous Traditional Chinese Medicine Inheritance Studio Construction Project in Guangdong Province (Guangdong Traditional Chinese Medicine Office [2019] No. 5), National Natural Science Foundation of China (No. 82174417), the Chinese Medicinal Scientific Research Project of Guangdong Province (No. 20211124), and Chinese Medicinal Scientific Research Project of Guangdong Province (No. 20211120).

## Conflict of Interest

The authors declare that the research was conducted in the absence of any commercial or financial relationships that could be construed as a potential conflict of interest.

## Publisher's Note

All claims expressed in this article are solely those of the authors and do not necessarily represent those of their affiliated organizations, or those of the publisher, the editors and the reviewers. Any product that may be evaluated in this article, or claim that may be made by its manufacturer, is not guaranteed or endorsed by the publisher.

## References

[1] Fetischeva LE Mozes VG Zakharov IS. ACOG practice bulletin No. 191 summary: tubal ectopic pregnancy. Obstet Gynecol. (2018) 131:409–11. 10.1097/AOG.0000000000002499 29370045

[2] Creanga AA Syverson C Seed K Callaghan WM. Pregnancy-related mortality in the United States, 2011–2013. Obstet Gynecol. (2017) 130:366–73. 10.1097/AOG.0000000000002114 28697109 PMC5744583

[3] Demirdag E Guler I Abay S Oguz Y Erdem M Erdem A. The impact of expectant management, systemic methotrexate and surgery on subsequent pregnancy outcomes in tubal ectopic pregnancy. Ir J Med Sci. (2017) 186:387–92. 10.1007/s11845-016-1419-5 26895299

[4] Edozien LC. Non-surgical management of ectopic pregnancy: appropriate risk management must be in place. Arch Gynecol Obstet. (2011) 283:925–7. 10.1007/s00404-010-1788-3 21153649

[5] Wu J Ludlow JP De Vries B Black K Beale P. Single-dose methotrexate treatment for ectopic pregnancy and pregnancy of unknown location and progesterone as a predictor of success. Aust N Z J Obstet Gynaecol. (2014) 54:469–74. 10.1111/ajo.12247 25287564

[6] Lipscomb GH Givens VA Meyer NL Bran D. Previous ectopic pregnancy as a predictor of failure of systemic methotrexate therapy. Fertil Steril. (2004) 81:1221–4. 10.1016/j.fertnstert.2003.09.070 15136080

[7] King AE Wheelhouse N Cameron S McDonald SE Lee KF Entrican G. Expression of secretory leukocyte protease inhibitor and elafin in human fallopian tube and in an *in-vitro* model of chlamydia trachomatis infection. Hum Reprod. (2009) 24:679–86. 10.1093/humrep/den452 19095674

[8] Eskicioglu F Özdemir AT Turan GA Gür EB Kasap E Genç M. The efficacy of complete blood count parameters in the diagnosis of tubal ectopic pregnancy. Ginekol Pol. (2014) 85:823–7. 10.17772/gp/1907 25675798

[9] Kan Ö Gemici A Alkilic A Cetindag EN Cakir C Dur R. The effect of preoperative neutrophil-to-lymphocyte ratio and platelet-to-lymphocyte ratio on predicting rupture risk in tubal ectopic pregnancies. Gynecol Obstet Invest. (2019) 84:378–82. 10.1159/000496543 30654361

[10] Kanmaz AG Inan AH Beyan E Budak A. Role of various complete blood count parameters in predicting the success of single-dose methotrexate in treating ectopic pregnancy. Pak J Med Sci. (2018) 34:1132–6. 10.12669/pjms.345.15356 30344563 PMC6191789

[11] Cekmez Y Göçmen A Sanlιkan F Türkmen SB. Role of mean platelet volume and neutrophil/lymphocyte ratio to predict single-dose methotrexate treatment success in tubal ectopic pregnancy. Clin Exp Obstet Gynecol. (2016) 43:509–11. 10.12891/ceog2152.2016 29734537

[12] Rabischong B Tran X Sleiman AA Larraín D Jaffeux P Aublet-Cuvelier B. Predictive factors of failure in management of ectopic pregnancy with single-dose methotrexate: a general population-based analysis from the auvergne register, France. Fertil Steril. (2011) 95:401–4. 10.1016/j.fertnstert.2010.08.025 20850718

[13] Bixby S Tello R Kuligowska E. Presence of a yolk sac on transvaginal sonography is the most reliable predictor of single-dose methotrexate treatment failure in ectopic pregnancy. J Ultrasound Med. (2005) 24:591–8. 10.7863/jum.2005.24.5.591 15840789

[14] Rekart ML Gilbert M Meza R Kim PH Chang M Money DM. Chlamydia public health programs and the epidemiology of pelvic inflammatory disease and ectopic pregnancy. J Infect Dis. (2013) 207:30–8. 10.1093/infdis/jis644 23100568

[15] Lee JH Kim S Lee I Yun J Yun BH Choi YS. A risk prediction model for medical treatment failure in tubal pregnancy. Eur J Obstet Gynecol Reprod Biol. (2018) 225:148–54. 10.1016/j.ejogrb.2018.04.020 29727784

[16] Sowter MC Farquhar CM Petrie KJ Gudex G. A randomised trial comparing single dose systemic methotrexate and laparoscopic surgery for the treatment of unruptured tubal pregnancy. BJOG. (2001) 108:192–203. 10.1111/j.1471-0528.2001.00038.x 11236120

[17] van Mello NM Mol F Ankum WM Mol BW van der Veen F Hajenius PJ. Ectopic pregnancy: how the diagnostic and therapeutic management has changed. Fertil Steril. (2012) 98:1066–73. 10.1016/j.fertnstert.2012.09.040 23084008

[18] von Elm E Altman DG Egger M Pocock SJ Gøtzsche PC Vandenbroucke JP. STROBE initiative. The strengthening the reporting of observational studies in epidemiology (STROBE) statement: guidelines for reporting observational studies. PLoS Med. (2007) 4:e296. 10.1371/journal.pmed.0040296 17941714 PMC2020495

[19] Domingue JN. Phenylpropanolamine contained in cold remedies and risk of hemorrhagic stroke. Neurology. (2007) 69:320–1. 10.1212/01.wnl.0000275291.02789.b3 17636076

[20] Akkaya H Uysal G. Can hematologic parameters predict treatment of ectopic pregnancy? Pak J Med Sci. (2017) 33:937–42. 10.12669/pjms.334.12418 29067069 PMC5648968

[21] Vandenbroucke JP von Elm E Altman DG Gøtzsche PC Mulrow CD Pocock SJ. STROBE initiative. strengthening the reporting of observational studies in epidemiology (STROBE): explanation and elaboration. PLoS Med. (2007) 4:e297. 10.1371/journal.pmed.0040297 17941715 PMC2020496

[22] Shaw JL Dey SK Critchley HO Horne AW. Current knowledge of the aetiology of human tubal ectopic pregnancy. Hum Reprod Update. (2010) 16:432–44. 10.1093/humupd/dmp057 20071358 PMC2880914

[23] Pisarska MD Carson SA Buster JE. Ectopic pregnancy. Lancet. (1998) 351:1115–20. 10.1016/S0140-6736(97)11476-3 9660597

[24] Tay JI Moore J Walker JJ. Ectopic pregnancy. West J Med. (2000) 173:131–4. 10.1136/ewjm.173.2.131 10924442 PMC1071024

[25] Balasubramaniam ES Van Noorden S El-Bahrawy M. The expression of interleukin (IL)-6, IL-8, and their receptors in fallopian tubes with ectopic tubal gestation. Fertil Steril. (2012) 98:898–904. 10.1016/j.fertnstert.2012.06.004 22763101

[26] Hvid M Baczynska A Deleuran B Fedder J Knudsen HJ Christiansen G. Interleukin-1 is the initiator of fallopian tube destruction during chlamydia trachomatis infection. Cell Microbiol. (2007) 9:2795–803. 10.1111/j.1462-5822.2007.00996.x 17614966

[27] Ji YF Chen LY Xu KH Yao JF Shi YF. Locally elevated leukemia inhibitory factor in the inflamed fallopian tube resembles that found in tubal pregnancy. Fertil Steril. (2009) 91:2308–14. 10.1016/j.fertnstert.2008.01.110 18468598

[28] Li P Zhu WJ Ma ZL Wang G Peng H Chen Y. Enhanced beta-catenin expression and inflammation are associated with human ectopic tubal pregnancy. Hum Reprod. (2013) 28:2363–71. 10.1093/humrep/det246 23787212

[29] King A Loke YW. On the nature and function of human uterine granular lymphocytes. Immunol Today. (1991) 12:432–5. 10.1016/0167-5699(91)90014-K 1786078

[30] Jones RL Hannan NJ Kaitu'u TJ Zhang J Salamonsen LA. Identification of chemokines important for leukocyte recruitment to the human endometrium at the times of embryo implantation and menstruation. J Clin Endocrinol Metab. (2004) 89:6155–67. 10.1210/jc.2004-0507 15579772

[31] Ramhorst R Grasso E Paparini D Hauk V Gallino L Calo G. Decoding the chemokine network that links leukocytes with decidual cells and the trophoblast during early implantation. Cell Adh Migr. (2016) 10:197–207. 10.1080/19336918.2015.1135285 26891097 PMC4853048

[32] Stewart-Akers AM Krasnow JS DeLoia JA. Decidual leukocyte populations in ectopic pregnancies. Fertil Steril. (1997) 68:1103–7. 10.1016/S0015-0282(97)00417-2 9418705

